# From information literacy to health literacy: AI-driven transformation in university libraries under digital public health—a perspective

**DOI:** 10.3389/fpubh.2026.1841351

**Published:** 2026-05-20

**Authors:** Ya-xin Sun, Li-ying Wu

**Affiliations:** Department of Library, Mudanjiang Medical University, Mudanjiang, China

**Keywords:** artificial intelligence, digital public health, health communication, health literacy, information literacy, university library

## Abstract

In the context of digital public health, the growing integration of artificial intelligence (AI) into health information environments challenges the adequacy of traditional information literacy frameworks in academic libraries. This perspective article argues for a transition from information literacy to AI-mediated health literacy education. It clarifies the conceptual distinctions between these two constructs, analyzes the AI-driven mechanisms reshaping educational paradigms, and identifies key institutional, technological, professional, and evaluative challenges. The article further proposes practical pathways that include mission reorientation, professional capacity building, platform development, collaborative governance, and evaluation system construction. This study advances existing literature by proposing an integrated, AI-mediated framework. This framework reconceptualizes health literacy within the context of digital public health. It also operationalizes this transformation through three interconnected dimensions: technological, educational, and governance-related. By outlining a theoretical framework and actionable strategies, it positions academic libraries as essential infrastructure for health literacy cultivation, contributing to health promotion and equity in an increasingly digital age.

## 1 Introduction

The rise of digital public health marks a paradigm shift in health governance, transitioning from a disease-centered model to one focused on comprehensive health management (1). Within this evolving landscape, health communication and health literacy have become essential components of public health infrastructure (1). As individuals navigate an increasingly mediated information environment, their ability to access, interpret, evaluate, and apply health-related information plays a critical role in shaping health inequities (2). Academic libraries have traditionally served as key sites for information literacy instruction, fostering students’ critical information skills (3, 4).

However, the growing integration of artificial intelligence (AI) into the production, distribution, and interactive dynamics of health information has challenged the adequacy of conventional information literacy frameworks (5, 6). These frameworks, which primarily emphasize search skills, are ill-equipped to address the complexities of an AI-infused health information environment characterized by algorithmic mediation, multimodal content, and the convergence of credible and misleading information (7). While conventional information literacy frameworks remain effective in relatively stable knowledge environments, they are predominantly grounded in cognitive models centered on information retrieval and evaluation (8). Such models insufficiently account for the algorithmically mediated epistemic conditions in which information visibility, credibility, and relevance are continuously shaped by opaque computational processes (9, 10). As a result, a critical conceptual limitation emerges: the absence of a context-sensitive and action-oriented literacy framework capable of linking informational comprehension with real-world health decision-making in AI-driven environments (8, 11). This disconnect calls for a fundamental reassessment of the educational role of academic libraries and a deliberate shift from generic information literacy toward a context-sensitive health literacy paradigm.

From a digital public health perspective, this article proposes a framework for transforming information literacy into health literacy within academic libraries through AI-mediated approaches. It advocates for the comprehensive integration of technological considerations, health-specific contexts, and pedagogical strategies to develop a literacy model that meets the demands of contemporary health governance. The perspective article aims to clarify the logic underlying this transformation and to outline practical pathways for its implementation. As a perspective article, it does not seek to present empirical evidence but rather to encourage theoretical reflection and critical dialogue among researchers and practitioners regarding the future direction of academic library education, thereby providing a conceptual foundation for further inquiry and practice.

## 2 Logical foundations of the transition: from information literacy to health literacy

### 2.1 Relationship and distinction between information literacy and health literacy

**Figure 1:**
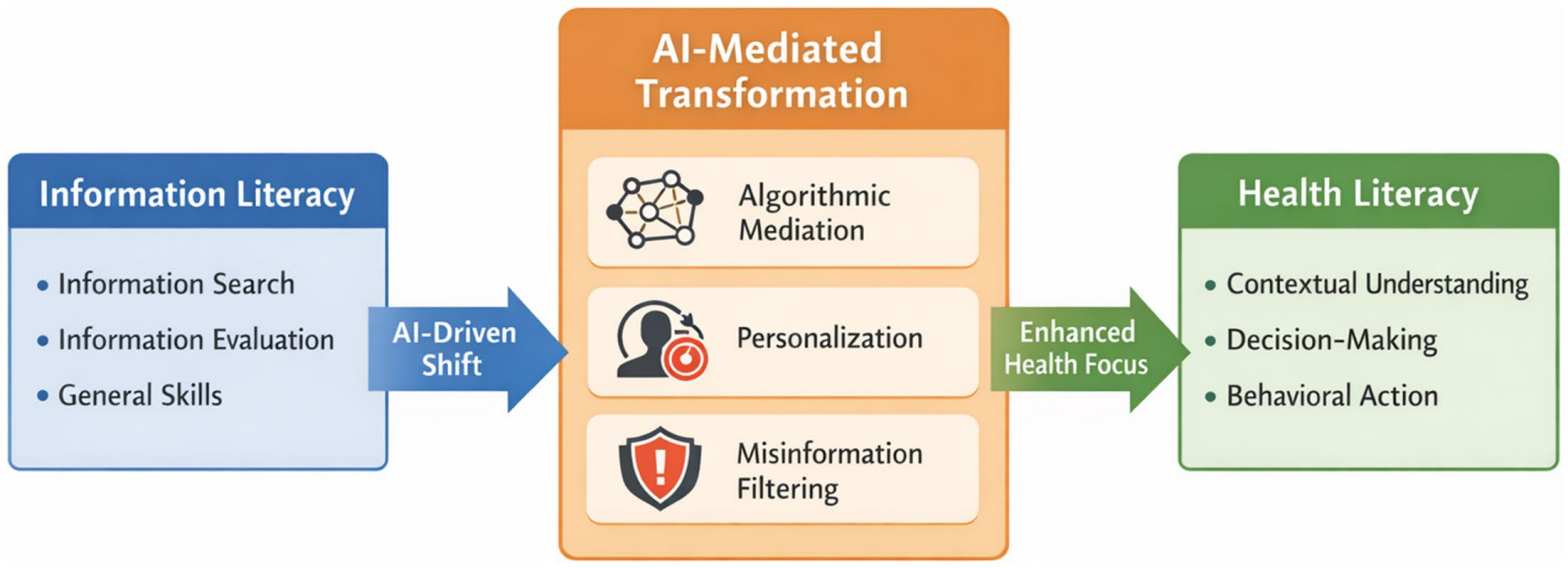
Conceptual transition from information literacy to Al-mediated health literacy.

A clear conceptual distinction between information literacy and health literacy is essential for understanding the rationale behind this transition. Information literacy traditionally emphasizes the ability to locate, retrieve, and evaluate information in general contexts (1). Health literacy, by contrast, extends beyond these cognitive skills to include the capacity to comprehend, critically appraise, and apply health-related information in ways that inform decisions and support health-promoting behaviors (12, 13). This distinction is especially important in an era marked by health information overload and the widespread proliferation of AI-generated content (14). The large volume and diversity of digital health information—much of which is algorithmically curated or synthetically produced—require not only evaluative skills but also contextual judgment and the ability to translate information into action (14). Health literacy therefore incorporates situational and behavioral dimensions that generic information literacy does not fully capture, making it a more suitable educational framework for navigating today’s complex health information environment (7) (Figure 1).

From a theoretical standpoint, health literacy can be further conceptualized as a multidimensional construct encompassing functional, interactive, and critical levels, which correspond to progressively higher degrees of cognitive processing and social engagement (8). In the context of AI-mediated information ecosystems, these dimensions undergo substantive transformation (9). Functional health literacy extends beyond basic comprehension to include the ability to navigate algorithmically curated and dynamically personalized content environments (10). Interactive health literacy increasingly involves engagement with digital interfaces, including conversational agents and intelligent recommendation systems, requiring users to interpret and respond to system-generated outputs (9, 10). At the highest level, critical health literacy entails the capacity to interrogate underlying algorithmic structures, assess data provenance, and evaluate the credibility and potential bias of AI-generated information (9). This theoretical reconfiguration underscores a shift from static information processing toward adaptive, context-sensitive competencies, thereby providing a more robust conceptual foundation for understanding health literacy in digital public health settings.

### 2.2 Evolution of digital public health communication

This conceptual shift reflects broader changes in digital public health communication. Traditional models of health communication were largely based on one-way transmission of expert knowledge (15). In the current digital landscape, communication has become increasingly interactive, participatory, and tailored to individual needs and circumstances (16, 17). This evolution places new demands on educational institutions, which should now equip individuals with the skills not only to receive information but also to engage with, contribute to, and critically assess health information across diverse platforms and formats (18).

Importantly, these transformations give rise to a reconfiguration of literacy competencies that extends beyond conventional communication paradigms (8, 11). Individuals are increasingly required to interpret algorithmically personalized health recommendations, critically evaluate AI-generated content in terms of reliability and bias, and synthesize multimodal information streams—including text, visual, and interactive formats—into context-sensitive health decisions (9–11, 19). Such competencies reflect a shift from passive information processing toward active, judgment-oriented engagement, thereby necessitating corresponding pedagogical innovation in curriculum design and instructional strategies (8, 19).

### 2.3 Redefining the role of university libraries

These developments require a fundamental redefinition of the academic library’s role. Historically positioned as an information intermediary, the library is now called upon to serve as a hub for health literacy cultivation (12). This shift addresses a key concern of digital public health: the social determinants of health (20). By embedding health literacy education into their core mission, academic libraries can help address the structural factors that shape health outcomes, including disparities in access, understanding, and agency (21, 22). In doing so, they move beyond a transactional model of information provision toward a more integrative and socially responsive educational function (23, 24). This reorientation forms the logical foundation for the AI-mediated transformation of literacy education explored in this study.

## 3 AI-driven mechanisms of transformation: technological integration and educational restructuring

### 3.1 AI as a catalyst for transformation

AI acts as a critical catalyst in the transition from information literacy to health literacy within academic libraries (25). Its applications span several domains essential to health information management, including intelligent content filtering, personalized recommendation systems, automated detection of misinformation, and AI-mediated behavioral interventions (26, 27). These capabilities not only reshape the information environment but also profoundly influence the content and delivery of literacy education (28). By automating aspects of information triage and personalization, AI shifts the educational focus from foundational retrieval skills toward higher-order competencies such as critical appraisal, contextual judgment, and informed decision-making in health contexts (28).

However, the integration of AI into health literacy education also introduces a set of structural and epistemic risks that warrant critical consideration (29, 30). Algorithmic bias embedded within data-driven systems may systematically distort information exposure, thereby reinforcing existing disparities in health knowledge access (29, 31). In addition, the opacity of algorithmic processes—often described as “black-box” decision-making—limits users’ ability to interrogate the provenance and reliability of recommended content (32, 33). Furthermore, excessive reliance on automated recommendations may attenuate learners’ independent evaluative capacities, potentially undermining the development of critical health literacy (30). Collectively, these limitations may compromise the trustworthiness of health information ecosystems and exacerbate existing inequities if not explicitly addressed within educational design and governance frameworks (31, 34).

### 3.2 Three dimensions of paradigm shift

This catalytic function gives rise to a threefold transformation in educational paradigms. First, in terms of content, instruction expands from generic information skills to include health information evaluation and health decision-making competencies (1, 12). This shift reflects the recognition that effective health literacy requires domain-specific knowledge and situational application. Second, with regard to methodology, education evolves from standardized training toward AI-assisted personalized learning pathways (35). Adaptive learning systems can tailor educational experiences to individual learners’ prior knowledge, skill gaps, and learning preferences, thereby enhancing engagement and outcomes (26, 35). For instance, AI-driven recommendation systems embedded within university library platforms can dynamically curate personalized health information resources based on users’ prior search behaviors and interaction patterns, thereby enabling more targeted and context-sensitive learning experiences (11, 19). Third, concerning context, educational delivery extends beyond traditional library services to achieve deeper integration with medical curricula, research processes, and community health initiatives (36). This contextual expansion embeds health literacy education into the academic and social environments where learners operate. In parallel, conversational agents and AI-enabled simulation tools can be employed to recreate health consultation scenarios, allowing learners to engage in interactive decision-making processes and apply acquired knowledge in practice-oriented contexts (37–39). Such implementations illustrate how the proposed three-dimensional framework can be operationalized within real-world educational settings, thereby enhancing both its practical relevance and pedagogical effectiveness (40, 41).

### 3.3 A data-driven closed loop in education

**Figure 2:**
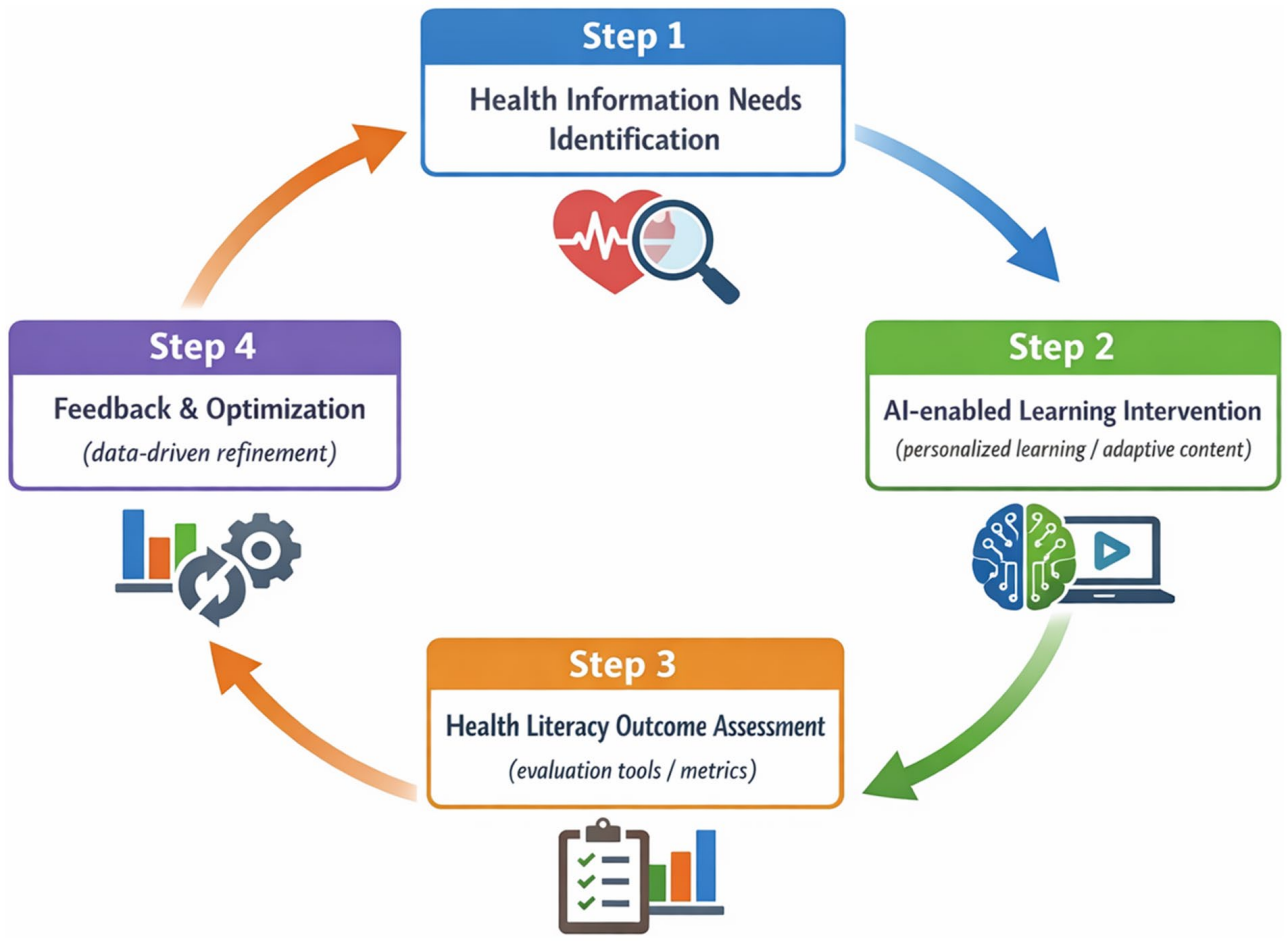
Al-driven closed-loop model for health literacy education in academic libraries.

Underpinning these transformations is a data-driven educational loop. By establishing a closed-loop mechanism that includes identifying health information needs, delivering AI-assisted learning interventions, and systematically assessing health literacy outcomes, academic libraries can adopt a more responsive and evidence-informed educational model (25, 42). This cyclical process enables continuous refinement of instructional strategies based on real-world performance data, ensuring that health literacy education remains adaptive, effective, and aligned with the evolving demands of digital public health (17, 43) (Figure 2).

To translate this conceptual model into practice, the operationalization of such closed-loop systems requires scalable data infrastructures capable of integrating heterogeneous learning and health-related data streams, alongside clearly defined governance frameworks to regulate data access, ownership, and accountability (44). This requirement becomes particularly critical in resource-constrained environments, where limitations in technical capacity and institutional support may hinder implementation and sustainability (45). Furthermore, embedding ethical principles into system design is essential to ensure responsible and trustworthy deployment; this includes safeguarding data privacy, establishing robust informed consent mechanisms, and promoting algorithmic transparency to mitigate risks associated with automated decision-making (44). Collectively, these considerations extend the closed-loop model from a purely pedagogical construct to an operational and ethically grounded framework suitable for real-world application.

## 4 Key challenges in the transition

The transition from information literacy to AI-mediated health literacy education in academic libraries faces multiple interrelated challenges across institutional, technological, professional, and evaluative dimensions.

### 4.1 Conceptual and organizational barriers

At the institutional level, conceptual and organizational barriers remain significant. The role of academic libraries in health literacy education has yet to be fully recognized within the broader university structure (46). Health literacy is often viewed as belonging to medical or public health schools, leaving libraries on the periphery (1, 12). This ambiguous position is worsened by the lack of established mechanisms for cross-departmental collaboration (1, 47). Without formal partnerships with academic departments, health services, and research units, libraries struggle to integrate health literacy instruction into the institutional framework in a sustained and scalable way (47).

Beyond these observable structural constraints, the barriers also reflect deeper governance-level challenges embedded within institutional systems (48, 49). Specifically, fragmented authority across administrative and academic units leads to unclear responsibility allocation, while competing disciplinary priorities hinder the alignment of shared educational objectives (48). In addition, the absence of effective incentive mechanisms—such as performance evaluation metrics or dedicated funding structures—further constrains cross-sector collaboration and limits the long-term sustainability of integrated health literacy initiatives (48). Collectively, these governance dynamics not only impede coordination but also weaken institutional capacity to operationalize interdisciplinary educational transformation (48).

### 4.2 Risks in AI technology application

Technological challenges arise from the use of AI in health education contexts. Algorithmic bias poses a significant risk, as AI systems may perpetuate or even amplify existing disparities in health information access and representation (50, 51). These risks extend beyond technical limitations and have systemic implications for health equity, as algorithmic decision-making processes may encode and reinforce pre-existing social and structural inequalities (52). Privacy concerns are equally pressing, especially regarding the collection and use of personal health data in learning environments (53). Inadequate data governance mechanisms may further exacerbate vulnerabilities, particularly for populations with limited digital literacy or reduced capacity to control personal data use (52).

In addition, the spread of AI-generated content raises fundamental questions about information authenticity, requiring learners to exercise greater critical judgment (54). Unequal access to AI-enabled educational resources may also contribute to a widening digital divide, whereby already disadvantaged groups face compounded barriers in acquiring reliable health information and developing critical evaluation skills (55). These technological risks introduce new ethical considerations that educational frameworks should explicitly address (26). Collectively, these dynamics may erode public trust in digital health systems, thereby undermining the effectiveness and legitimacy of AI-mediated health education initiatives if not proactively addressed through equitable design and governance strategies (56, 57).

### 4.3 Insufficient professional competencies of librarians

The professional capacity of library staff represents another critical challenge. Effective health literacy education requires a combination of competencies that go beyond traditional librarianship (47, 58). These include foundational knowledge in health sciences, proficiency in AI technologies, and skills in health communication (59). The current shortage of professionals with such interdisciplinary expertise limits the scope and quality of health literacy initiatives (58, 60). Addressing this gap requires structured training pathways, interdisciplinary curricula, and institutional investment in continuous professional development. These elements are necessary to sustain competency transformation.

### 4.4 Absence of health literacy assessment frameworks

Finally, the absence of robust evaluation frameworks hinders progress. There is a notable lack of validated assessment tools designed specifically to measure health literacy outcomes in higher education settings (1). Without reliable instruments to assess learning effectiveness, institutions struggle to demonstrate impact, secure support, and refine instructional approaches (2, 61). Addressing these multifaceted challenges is essential to realizing the full potential of AI-driven health literacy education in academic libraries.

## 5 Practical pathways for digital public health

Realizing the transition from information literacy to AI-mediated health literacy education requires a coherent set of practical pathways addressing institutional strategy, professional development, resource infrastructure, collaborative governance, and evaluation mechanisms. To enhance practical applicability, each pathway can be operationalized through clearly defined stakeholders, implementation strategies, and measurable outcomes.

### 5.1 Pathway one: redefining institutional mission

The first step is to embed health literacy into the core mission of academic libraries (62). This involves a strategic shift from viewing the library as an information center to establishing it as a hub for health literacy cultivation (63). Such reorientation requires institutional recognition, leadership commitment, and alignment with broader university priorities related to health and well-being.

### 5.2 Pathway two: rebuilding professional competencies

Effective implementation depends on a reconfigured workforce. A competency framework combining librarianship, health sciences, and AI literacy should be developed to guide professional development (64, 65). Creating dedicated positions focused on health literacy education can help institutionalize this function and attract interdisciplinary talent (64, 66).

### 5.3 Pathway three: developing resources and AI-driven platforms

Developing specialized infrastructure is essential. This includes curating authoritative health information repositories and designing AI-enabled learning platforms that deliver personalized, adaptive support (26). Such platforms can tailor content to individual learners’ needs, track progress, and provide timely interventions, thereby enhancing engagement and learning outcomes (67, 68). The successful development and implementation of these platforms require the coordinated involvement of key stakeholders, including library administrators, information technology specialists, public health experts, and academic faculty (69). The effectiveness of such infrastructure should be evaluated through measurable outcomes, encompassing user engagement metrics, health literacy assessment scores, and system usability indicators (19).

### 5.4 Pathway four: fostering collaborative governance

No single unit can accomplish this transition alone. A networked governance structure involving libraries, schools of public health, university health services, and student affairs departments is needed to form a health literacy education consortium (1, 70). This collaborative model enables resource sharing, curriculum integration, and coordinated outreach, ensuring that health literacy education is both comprehensive and sustainable (36).

### 5.5 Pathway five: establishing evaluation systems

Progress should be measured with appropriate assessment tools. Developing validated instruments to evaluate health literacy outcomes in higher education settings is a priority (71, 72). Moreover, integrating health literacy into the broader student competency assessment framework signals institutional commitment and encourages learner engagement (2, 73). Together, these pathways offer a systematic approach to advancing health literacy education in alignment with the principles and demands of digital public health (Supplementary Table 1).

## 6 Summary

This perspective article has argued that the transition from information literacy to AI-mediated health literacy education in academic libraries is both a necessary response to the evolving landscape of digital public health communication and a key direction for the innovation of library functions. As AI increasingly shapes the production, dissemination, and consumption of health information, traditional information literacy frameworks are no longer sufficient to equip learners with the competencies needed to navigate this complex environment. Health literacy, with its focus on contextual judgment, decision-making, and behavioral application, offers a more suitable educational paradigm.

By outlining the logical foundations of this transition, examining AI-driven mechanisms of transformation, identifying key challenges, and proposing practical pathways, this paper provides a conceptual framework for reimagining the role of academic libraries within the digital public health ecosystem. This framework makes a unique contribution by integrating AI-mediated mechanisms with a health literacy paradigm within academic library systems. It offers a structured yet adaptable model for digital public health education. The analysis offers insights for institutional policy development, educational reform, and professional capacity building.

Looking forward, there is a pressing need for interdisciplinary research to explore best practices in AI-enhanced health literacy education. Future research should focus on empirical validation and cross-institutional implementation models. It should also aim to develop standardized evaluation tools that can help translate this conceptual framework into scalable practice. Such efforts should aim to establish academic libraries as essential infrastructure for public health communication, positioning them to contribute meaningfully to health promotion and health equity in an increasingly digital age.

## Data Availability

The original contributions presented in the study are included in the article/Supplementary material, further inquiries can be directed to the corresponding author.

## References

[1] Sørensen K Van den Broucke S Fullam J Doyle G Pelikan J Slonska Z . Health literacy and public health: a systematic review and integration of definitions and models. BMC Public Health. (2012) 12:80. doi: 10.1186/1471-2458-12-80, 22276600 PMC3292515

[2] Nutbeam D. The evolving concept of health literacy. Soc Sci Med. (2008) 67:2072–8. doi: 10.1016/j.socscimed.2008.09.050 18952344

[3] Bawden D Robinson L. The dark side of information: overload, anxiety and other paradoxes and pathologies. J Inf Sci. (2009) 35:180–91. doi: 10.1177/0165551508095781

[4] Diviani N van den Putte B Giani S van Weert JC. Low health literacy and evaluation of online health information: a systematic review of the literature. J Med Internet Res. (2015) 17:e112. doi: 10.2196/jmir.4018, 25953147 PMC4468598

[5] Mesko B. The role of artificial intelligence in precision medicine. Expert Rev Precis Med Drug Dev. (2017) 2:239–41. doi: 10.1080/23808993.2017.1380516

[6] Nutbeam D Milat AJ. Artificial intelligence and public health: prospects, hype and challenges. Public Health Res Pract. (2025) 35:PU24001. doi: 10.1071/PU24001 40443074

[7] Olawade DB Wada OJ David-Olawade AC Kunonga E Abaire O Ling J. Using artificial intelligence to improve public health: a narrative review. Front Public Health. (2023) 11:1196397. doi: 10.3389/fpubh.2023.1196397, 37954052 PMC10637620

[8] Ban S Kim Y Seomun G. Digital health literacy: a concept analysis. Digit Health. (2024) 10:20552076241287894. doi: 10.1177/20552076241287894, 39381807 PMC11459536

[9] Lopez-Lopez E Abels CM Holford D Herzog SM Lewandowsky S. Generative artificial intelligence-mediated confirmation bias in health information seeking. Ann N Y Acad Sci. (2025) 1550:23–36. doi: 10.1111/nyas.15413, 40716036 PMC12412720

[10] Luo Y Zhao Z Xu X Zhao Y Yang F. The influence of recommendation algorithms on users' intention to adopt health information: does trust belief play a role? J Am Med Inform Assoc. (2025) 32:1415–24. doi: 10.1093/jamia/ocaf115, 40680291 PMC12361861

[11] Tilton AK Caplan BE Cole BJ. Generative AI in consumer health: leveraging large language models for health literacy and clinical safety with a digital health framework. Front Digit Health. (2025) 7:1616488. doi: 10.3389/fdgth.2025.1616488, 40933812 PMC12417475

[12] Nutbeam D. Health literacy as a public health goal: a challenge for contemporary health education and communication strategies into the 21st century. Health Promot Int. (2000) 15:259–67. doi: 10.1093/heapro/15.3.259

[13] Kim H Xie B. Health literacy in the eHealth era: a systematic review of the literature. Patient Educ Couns. (2017) 100:1073–82. doi: 10.1016/j.pec.2017.01.015, 28174067

[14] Jacobs RJ Lou JQ Ownby RL Caballero J. A systematic review of eHealth interventions to improve health literacy. Health Informatics J. (2016) 22:81–98. doi: 10.1177/1460458214534092 24916567

[15] Ventola CL. Social media and health care professionals: benefits, risks, and best practices. P T. (2014) 39:491–520. 25083128 PMC4103576

[16] Moorhead SA Hazlett DE Harrison L Carroll JK Irwin A Hoving C. A new dimension of health care: systematic review of the uses, benefits, and limitations of social media for health communication. J Med Internet Res. (2013) 15:e85. doi: 10.2196/jmir.1933, 23615206 PMC3636326

[17] Kreps GL Neuhauser L. New directions in eHealth communication: opportunities and challenges. Patient Educ Couns. (2010) 78:329–36. doi: 10.1016/j.pec.2010.01.013, 20202779

[18] Chou WS Oh A Klein WMP. Addressing health-related misinformation on social media. JAMA. (2018) 320:2417–8. doi: 10.1001/jama.2018.16865 30428002

[19] Abeo ANA Armstrong S Scriney M Goss H. Artificial intelligence techniques and health literacy: a systematic review. Mayo Clin Proc Digit Health. (2025) 3:100269. doi: 10.1016/j.mcpdig.2025.100269, 41211528 PMC12589913

[20] Berkman ND Sheridan SL Donahue KE Halpern DJ Crotty K. Low health literacy and health outcomes: an updated systematic review. Ann Intern Med. (2011) 155:97–107. doi: 10.7326/0003-4819-155-2-201107190-00005 21768583

[21] Luo L Park VT. Health literacy and libraries: a literature review. Ref Serv Rev. (2016) 44:191–205. doi: 10.1108/RSR-02-2016-0005

[22] Sentell T Vamos S Okan O. Interdisciplinary perspectives on health literacy research around the world: more important than ever in a time of COVID-19. Int J Environ Res Public Health. (2020) 17:3010. doi: 10.3390/ijerph17093010, 32357457 PMC7246523

[23] Logan RA Siegel ER. Health Literacy: New Directions in Research, Theory and Practice. Amsterdam, Netherlands: IOS Press (2017).

[24] Pleasant A Cabe J Patel K Cosenza J Carmona R. Health literacy research and practice: a needed paradigm shift. Health Commun. (2015) 30:1176–80. doi: 10.1080/10410236.2015.1037426 26372030

[25] Bickmore TW Trinh H Olafsson S O'Leary TK Asadi R Rickles NM . Patient and consumer safety risks when using conversational assistants for medical information: an observational study of Siri, Alexa, and Google assistant. J Med Internet Res. (2018) 20:e11510. doi: 10.2196/11510, 30181110 PMC6231817

[26] Topol EJ. High-performance medicine: the convergence of human and artificial intelligence. Nat Med. (2019) 25:44–56. doi: 10.1038/s41591-018-0300-7, 30617339

[27] Wang Y McKee M Torbica A Stuckler D. Systematic literature review on the spread of health-related misinformation on social media. Soc Sci Med. (2019) 240:112552. doi: 10.1016/j.socscimed.2019.112552, 31561111 PMC7117034

[28] Luckin R Holmes W Griffiths M Forcier LB. Intelligence Unleashed: An Argument for AI in Education. London: Pearson (2016).

[29] Hasanzadeh F Josephson CB Waters G Adedinsewo D Azizi Z White JA. Bias recognition and mitigation strategies in artificial intelligence healthcare applications. NPJ Digit Med. (2025) 8:154. doi: 10.1038/s41746-025-01503-7, 40069303 PMC11897215

[30] Cao Y Deng L Liu X Feng Z Gao Y. Ethical challenges in the algorithmic era: a systematic rapid review of risk insights and governance pathways for nursing predictive analytics and early warning systems. BMC Med Ethics. (2025) 26:151. doi: 10.1186/s12910-025-01308-z, 41168748 PMC12574269

[31] Cross JL Choma MA Onofrey JA. Bias in medical AI: implications for clinical decision-making. PLOS Digit Health. (2024) 3:e0000651. doi: 10.1371/journal.pdig.0000651, 39509461 PMC11542778

[32] Nouis SC Uren V Jariwala S. Evaluating accountability, transparency, and bias in AI-assisted healthcare decision-making: a qualitative study of healthcare professionals' perspectives in the UK. BMC Med Ethics. (2025) 26:89. doi: 10.1186/s12910-025-01243-z, 40629303 PMC12235780

[33] Allen JW Wilkinson D Savulescu J. When is it safe to introduce an AI system into healthcare? A practical decision algorithm for the ethical implementation of black-box AI in medicine. Bioethics. (2026) 40:61–72. doi: 10.1111/bioe.70032, 40964933 PMC12710678

[34] Gao Q Chen L Huang Z. Opportunities and challenges of artificial intelligence in public health: a systematic review on technological efficacy, ethical dilemmas, and governance pathways. Front Public Health. (2026) 13:1748797. doi: 10.3389/fpubh.2025.1748797, 41613098 PMC12847321

[35] Khalil MK Elkhider IA. Applying learning theories and instructional design models for effective instruction. Adv Physiol Educ. (2016) 40:147–56. doi: 10.1152/advan.00138.2015 27068989

[36] Rowlands G Shaw A Jaswal S Smith S Harpham T. Health literacy and the social determinants of health: a qualitative model from adult learners. Health Promot Int. (2017) 32:dav093–138. doi: 10.1093/heapro/dav093, 28180257

[37] Holderried F Stegemann-Philipps C Herrmann-Werner A Festl-Wietek T Holderried M Eickhoff C . A language model-powered simulated patient with automated feedback for history taking: prospective study. JMIR Med Educ. (2024) 10:e59213. doi: 10.2196/59213, 39150749 PMC11364946

[38] Lee HY Kim J Choi H Bae H Jeong A Choi S . Comparing AI chatbot simulation and peer role-play for OSCE preparation: a pilot randomized controlled trial. BMC Med Educ. (2025) 25:1755. doi: 10.1186/s12909-025-08308-y, 41286823 PMC12750692

[39] Zhong D Chow SKK. Investigating the potential of generative AI clinical case-based simulations on radiography education: a pilot study. J Imaging Inform Med. (2026) 39:1848–60. doi: 10.1007/s10278-025-01601-8, 40627294 PMC13103059

[40] Gilbert V Philip P Llorca PM Samalin L. Use of artificial intelligence-enhanced virtual patients in educational approaches to medical interview training: a systematic review. BMC Med Educ. (2026) 26:588. doi: 10.1186/s12909-026-08804-9, 41781962 PMC13067467

[41] Cheng A McGregor C. Applications of artificial intelligence in healthcare simulation: a model of thinking. Adv Simul. (2025) 10:45. doi: 10.1186/s41077-025-00379-7, 40968387 PMC12447618

[42] Bickmore T Giorgino T. Health dialog systems for patients and consumers. J Biomed Inform. (2006) 39:556–71. doi: 10.1016/j.jbi.2005.12.004, 16464643

[43] Murray E Hekler EB Andersson G Collins LM Doherty A Hollis C . Evaluating digital health interventions: key questions and approaches. Am J Prev Med. (2016) 51:843–51. doi: 10.1016/j.amepre.2016.06.008, 27745684 PMC5324832

[44] Mahesh N Devishamani CS Raghu K Mahalingam M Bysani P Chakravarthy AV . Advancing healthcare: the role and impact of AI and foundation models. Am J Transl Res. (2024) 16:2166–79. doi: 10.62347/WQWV9220, 39006256 PMC11236664

[45] Green BL Murphy A Robinson E. Accelerating health disparities research with artificial intelligence. Front Digit Health. (2024) 6:1330160. doi: 10.3389/fdgth.2024.1330160, 38322109 PMC10844447

[46] Ma J Stahl L Knotts E. Emerging roles of health information professionals for library and information science curriculum development: a scoping review. J Med Libr Assoc. (2018) 106:432–44. doi: 10.5195/jmla.2018.354, 30271284 PMC6148628

[47] Shipman JP Kurtz-Rossi S Funk CJ. The health information literacy research project. J Med Libr Assoc. (2009) 97:293–301. doi: 10.3163/1536-5050.97.4.014, 19851494 PMC2759165

[48] Sørensen K Levin-Zamir D Duong TV Okan O Brasil VV Nutbeam D. Building health literacy system capacity: a framework for health literate systems. Health Promot Int. (2021) 36:i13–23. doi: 10.1093/heapro/daab153, 34897445 PMC8672927

[49] Nutbeam D Lloyd JE. Understanding and responding to health literacy as a social determinant of health. Annu Rev Public Health. (2021) 42:159–73. doi: 10.1146/annurev-publhealth-090419-102529 33035427

[50] Obermeyer Z Powers B Vogeli C Mullainathan S. Dissecting racial bias in an algorithm used to manage the health of populations. Science. (2019) 366:447–53. doi: 10.1126/science.aax2342, 31649194

[51] Rajkomar A Hardt M Howell MD Corrado G Chin MH. Ensuring fairness in machine learning to advance health equity. Ann Intern Med. (2018) 169:866–72. doi: 10.7326/M18-1990, 30508424 PMC6594166

[52] Acosta JA. Perspective: advancing public health education by embedding AI literacy. Front Digit Health. (2025) 7:1584883. doi: 10.3389/fdgth.2025.1584883, 40741324 PMC12307283

[53] Price WN 2nd Cohen IG. Privacy in the age of medical big data. Nat Med. (2019) 25:37–43. doi: 10.1038/s41591-018-0272-7, 30617331 PMC6376961

[54] Blease C Kaptchuk TJ Bernstein MH Mandl KD Halamka JD DesRoches CM. Artificial intelligence and the future of primary care: exploratory qualitative study of UK general practitioners' views. J Med Internet Res. (2019) 21:e12802. doi: 10.2196/12802, 30892270 PMC6446158

[55] Dankwa-Mullan I Ndoh K Akogo D Rocha HAL Juaçaba SF. Artificial intelligence and Cancer health equity: bridging the divide or widening the gap. Curr Oncol Rep. (2025) 27:95–111. doi: 10.1007/s11912-024-01627-1 39753817

[56] Zondag AGM Rozestraten R Grimmelikhuijsen SG Jongsma KR van Solinge WW Bots ML . The effect of artificial intelligence on patient-physician trust: Cross-sectional vignette study. J Med Internet Res. (2024) 26:e50853. doi: 10.2196/50853, 38805702 PMC11167322

[57] Jain A Salas M Aimer O Adenwala Z. Safeguarding patients in the AI era: ethics at the forefront of pharmacovigilance. Drug Saf. (2025) 48:119–27. doi: 10.1007/s40264-024-01483-9 39331228

[58] Detlefsen EG. The education of informationists, from the perspective of a library and information sciences educator. J Med Libr Assoc. (2002) 90:59–67. 11838461 PMC64758

[59] Zhang Y Sun Y Xie B. Quality of health information for consumers on the web: a systematic review of indicators, criteria, tools, and evaluation results. J Assoc Inf Sci Technol. (2015) 66:2071–84. doi: 10.1002/asi.23311

[60] Dalmer NK. Questioning reliability assessments of health information on social media. J Med Libr Assoc. (2017) 105:61–8. doi: 10.5195/jmla.2017.108, 28096748 PMC5234445

[61] Tudor Car L Poon S Kyaw BM Cook DA Ward V Atun R . Digital education for health professionals: an evidence map, conceptual framework, and research agenda. J Med Internet Res. (2022) 24:e31977. doi: 10.2196/31977, 35297767 PMC8972116

[62] Whitney W Keselman A Humphreys B. Libraries and librarians: key Partners for Progress in health literacy research and practice. Stud Health Technol Inform. (2017) 240:415–32. doi: 10.3233/ISU-170821, 28972531 PMC5724359

[63] Naccarella L Horwood J. Public libraries as health literate multi-purpose workspaces for improving health literacy. Health Promot J Austr. (2021) 32:29–32. doi: 10.1002/hpja.437, 33140444

[64] Wamala-Andersson S Uitto M Diop-Christensen A Hidalgo IR Thorup CB Heß F . Understanding digital health literacy as a structural determinant of health and public health capability. BMC Glob Public Health. (2026) 4:7. doi: 10.1186/s44263-025-00236-9, 41535922 PMC12805707

[65] Car J Ong QC Erlikh Fox T Leightley D Kemp SJ Švab I . The digital health competencies in medical education framework: an international consensus statement based on a Delphi study. JAMA Netw Open. (2025) 8:e2453131. doi: 10.1001/jamanetworkopen.2024.53131, 39888625

[66] Boshnjaku A Krasniqi E Kamberi F. The emerging need to integrate digital health literacy as a course into health-related and care-related profession curricula. Front Public Health. (2025) 13:1534139. doi: 10.3389/fpubh.2025.1534139 40066010 PMC11891342

[67] Triantafyllidis AK Tsanas A. Applications of machine learning in real-life digital health interventions: review of the literature. J Med Internet Res. (2019) 21:e12286. doi: 10.2196/12286, 30950797 PMC6473205

[68] Laranjo L Dunn AG Tong HL Kocaballi AB Chen J Bashir R . Conversational agents in healthcare: a systematic review. J Am Med Inform Assoc. (2018) 25:1248–58. doi: 10.1093/jamia/ocy072, 30010941 PMC6118869

[69] Izquierdo-Condoy JS Arias-Intriago M Montero Corrales L Ortiz-Prado E. Artificial intelligence in medical education: transformative potential, current applications, and future implications. JMIR Med Educ. (2026) 12:e77127. doi: 10.2196/77127, 41701936 PMC12912660

[70] Kickbusch I Pelikan JM Apfel F Tsouros AD. Health Literacy: the Solid Facts. Copenhagen: World Health Organization (2013).

[71] Sørensen K Van den Broucke S Pelikan JM Fullam J Doyle G Slonska Z . Measuring health literacy in populations: illuminating the design and development process of the European health literacy survey questionnaire (HLS-EU-Q). BMC Public Health. (2013) 13:948. doi: 10.1186/1471-2458-13-948, 24112855 PMC4016258

[72] Haun JN Valerio MA McCormack LA Sørensen K Paasche-Orlow MK. Health literacy measurement: an inventory and descriptive summary of 51 instruments. J Health Commun. (2014) 19:302–33. doi: 10.1080/10810730.2014.936571, 25315600

[73] Coleman CA Hudson S Maine LL. Health literacy practices and educational competencies for health professionals: a consensus study. J Health Commun. (2013) 18:82–102. doi: 10.1080/10810730.2013.829538, 24093348 PMC3814998

