# Supplementary material for: From information literacy to health literacy: AI-driven transformation in university libraries under digital public health—a perspective
Source: Front Public Health. 2026 May 20;14:1841351. doi: 10.3389/fpubh.2026.1841351 (PMC13229875; doi:10.3389/fpubh.2026.1841351)
Supplement: Supplementary file 1 [file Table_1.DOCX]

**Supplementary Table 1. Practical Pathways for AI-Mediated Health Literacy Transformation in Academic Libraries**

| **Pathway** | **Core objective** | **Key actions** | **Stakeholders** | **Expected impact** |
| --- | --- | --- | --- | --- |
| Pathway 1 | Redefining mission | Embed health literacy into strategy | Library leadership | Institutional alignment |
| Pathway 2 | Workforce development | Build interdisciplinary competencies | Librarians, educators | Capacity enhancement |
| Pathway 3 | Infrastructure | AI platforms &  repositories | IT + library | Personalized learning |
| Pathway 4 | Governance | Cross-sector collaboration | Public health,  schools | Sustainability |
| Pathway 5 | Evaluation | Develop assessment tools | Researchers, institutions | Measurable outcomes |

**Abbreviations**: AI, artificial intelligence; IT, information technology.
